# Manufacturing sector spatial pattern evolution and its relationship with regional economic differences: Evidence from Jiangsu, China

**DOI:** 10.1371/journal.pone.0312135

**Published:** 2024-11-20

**Authors:** Enkang Li, Zhifeng Liu, Yingyi Ma, Wen Zhong, Ruoyan Zhang

**Affiliations:** Jinling Institute of Technology, School of Architectural Engineering, Nanjing, China; The Chinese University of Hong Kong, HONG KONG

## Abstract

This study presents the case of China’s Jiangsu Province. The spatial-temporal pattern evolution of different manufacturing sectors is discussed using spatial analysis technology (spatial autocorrelation and standard deviation ellipses). The Granger test is used to analyze the relationship between the change in the manufacturing industry spatial agglomeration and regional economic differences. The following conclusions are drawn: 1) The spatial agglomeration trend of most manufacturing sectors is weakening. Much of the manufacturing sector, like the rubber and plastic product industries, has been transferred from southern to northern Jiangsu. 2) From the scale, only a minority of these enterprises possess substantial registered capital. The capital injection scale of more manufacturing enterprises is insignificant. At the same time, manufacturing companies with substantial financial resources are increasingly inclined to choose less-concentrated areas when choosing new investment areas. 3) The reduction of regional economic differences is considered to be the Granger-cause for the decline of the spatial agglomeration degree of the manufacturing industry in Jiangsu Province. Analyzing the spatiotemporal pattern of the manufacturing industry in Jiangsu Province will provide specific policy reference values for the manufacturing industry and economic development of Jiangsu province. In addition, for companies of different sizes, the findings of this paper also provide valuable references on how they can choose suitable investment locations according to their size in the future.

## Introduction

Industrial agglomeration is this era’s most prominent economic trend [1,2]. As one of the essential industrial categories, manufacturing has promoted rapid regional economic growth through spatial agglomeration [3–5].

Jiangsu is an economically developed province along China’s east coast. It is a critical Yangtze River Delta region (YRD) member. Since China’s reform and opening up, Jiangsu’s manufacturing has flourished. In 2020, the number of manufacturing enterprises above the designated size in Jiangsu Province was 49,280, with a total profit of 712.606 billion yuan. The three most profitable types of manufacturing are computers, communications, and other electronic equipment; electrical machinery and equipment; and general equipment. This situation is primarily due to the economic benefits of enterprise spatial agglomeration, which has been consistently demonstrated in many prominent research studies from work by Marshall until the present time [6,7]. For instance, Brave and Mattoon argue that the degree of complementarity of a Metropolitan Statistical Area (MSA) industry clusters can lead to its persistent economic advantage over its peers, stemming primarily from the level of integration or Co‐agglomeration of an MSA’s clusters [8].

The manufacturing industry is crucial to Jiangsu and YRD’s economic development. However, problems exist that have yet to be addressed by researchers.

In China, the county’s prosperity significantly affects provincial economic development. The choice of districts and counties where new enterprises in different manufacturing sectors invest and build factories is related to the spatial coordination of each province’s economy. Since the start of the 21st century, China has gone from being a non-member to an essential member of the WTO. It has faced various challenges, including the financial crisis, the China–US trade war, and the COVID-19 pandemic. Jiangsu’s manufacturing industry’s spatial and temporal pattern has changed substantially in this process. Monitoring and analyzing this change in different sectors could reveal future development countermeasures for Jiangsu’s manufacturing industries. Second, in addition to specific commonalities in spatial layout, particular outcomes should be examined when different manufacturing sectors establish new factories. We must find ways to characterize their performance quantitatively. For example, we want to know whether a larger business, when established, would choose not to cluster in the same county as a smaller business. Third, examining the relationship between the change of spatial layout of the manufacturing industry and regional economic differences is important. If we clearly understand the relationship between them, useful suggestions can be made for the future economic development of Jiangsu.

The above three problems are the focus of this paper.

## Literature review

Before we formally introduce our research, we would like to overview the literature and the scholarly views it conveys. Our literature review examines two aspects. The first issue concerns how the research on manufacturing agglomeration and spatial distribution is performed from the perspectives of economics and geography.

### The economics of manufacturing agglomeration: An economic perspective

From an economic perspective, the degree of manufacturing agglomeration is related to the efficiency of regional growth, urbanization, regional innovation, and the urban and rural ecological environment [9,10]. Economists have proposed different answers to the question of potentially beneficial agglomeration.

First, moderate agglomeration’s ability to unleash significant economic dynamism is widely acknowledged [11,12]. Wang’s study revealed differences concerning the optimal agglomeration size of capital/technology and labor-intensive industries [13]. Excessive agglomeration may lead to production efficiency decline and environmental problems [14,15].

Second, the economic growth provided by agglomeration is unequal across industrial categories. In general, within a city, the strength of the agglomeration effects of different economic activities varies due to the sector-specific nature of required resources, human capital, and exceptional production and technology factors [16]. Delgado believes that industries with different knowledge intensity levels gain various economic benefits from agglomeration [17]. Faggio and Hervas-Oliver also believe that the economic effects of agglomeration vary for different enterprises and industries [18,19].

Third, distance plays an essential role in agglomeration. Consensus exists that more firms and a denser economy are found in the core area of agglomeration. The closer the firm is to the core, the easier knowledge spillovers occur due to agglomeration [20–22].

However, agglomeration is not a panacea. It only plays a role when the economic level develops to a particular stage [23]. Clusters of industries that complement rather than compete with each other will drive the economy to a new level [24]. This economic boost is often reflected in productivity improvement. Many researchers, including Cai and Najkar, have positive attitudes toward improving productivity by agglomeration [25,26]. However, Wang examined the Beijing-Tianjin-Hebei region in China and found that manufacturing agglomeration would bring about a total factor productivity (TFP) decline [27].

### Spatial characteristics of manufacturing agglomeration: A geographical perspective

From a geographical perspective, the manufacturing agglomeration discussion focuses more on spatial pattern changes, such as the interannual change of specific attributes (including the number of employees, output value, investment, and other aspects) of the same manufacturing sector within a specific geographical scope. Therefore, exploratory spatial data analysis (ESDA) is a crucial technology for describing the spatial patterns of manufacturing industries.

Undoubtedly, spatial autocorrelation is one of the most powerful tools for studying the spatial pattern of the manufacturing industry because of its ease of operation and the reliability of spatial data analysis. For instance, Hassan et al. studied the characteristics of the spatial autocorrelation of six manufacturing industries, including brick, food, garments, machinery, metal, and plastic. They found that the spatial distribution of all manufacturing industries tends toward non-randomness, given observed and expected values [28]. The differences in the spatial distribution across the sectors are also apparent. Guillain and Le Gallo demonstrated great diversity in location patterns across sectors using Moran scatterplots and local statistical indicators of spatial association [29].

Bivariate spatial correlation is also widely used for applying spatial autocorrelation with the classical Moran’s I of unary variables [30,31]. The primary advantage of bivariate spatial correlation is that it facilitates an analysis of associations between the spatial agglomeration or dispersion of manufacturing industries and patterns of other economic factors. For example, Cheng analyzed the spatial correlation and interaction between manufacturing agglomeration and environmental pollution using global bivariate Moran’s I [32]. Debnath and Naznin also used global bivariate Moran’s I. They found a spatial dependence relationship between manufacturing agglomeration and slum distribution [33].

Scale selection is another concern in applying spatial autocorrelation analysis of manufacturing agglomeration. For example, China’s spatial autocorrelation analysis of manufacturing agglomeration involves the provincial [34] and county scales [35]. The latter has more abundant spatial information than the former. However, regardless of the scale, the spatial autocorrelation analysis results show that the manufacturing industry and regional development have strong path dependence and spatial locking characteristics [36,37].

### Summary of the literature

Research on the spatial distribution of the manufacturing industry has been rich from the perspective of economics and geography. However, existing literature has insufficiently investigated certain problems. First, comparative analyses of the spatial patterns of different types of manufacturing industries are limited. Second, a lack of correlation analysis between the scale difference and spatial pattern of manufacturing enterprises persists. Third, the integrated research on the manufacturing industry from the dual perspectives of geography and economics is insufficient, and the relationship between the spatial distribution of the manufacturing industry and regional economic differences needs to be empirically analyzed.

## Materials and methods

### Spatial autocorrelation

Spatial autocorrelation is essential for analyzing industrial agglomeration [38–40]. The formula for Moran’s I statistic for spatial autocorrelation is:

$$I=\frac{n}{S_{0}}\frac{\sum_{i=1}^{n}\sum_{j=1}^{n}w_{i,j}z_{i}z_{j}}{\sum_{i=1}^{n}{z_{i}}^{2}}$$ (1)

where *z*_*i*_ is the deviation of an attribute for feature *i* from its mean $\left(x_{i}-\bar{X}\right)$, *w*_*i*,*j*_ is the spatial weight between feature *i* and *j*; *n* is equal to the total number of features, and *S*_0_ is the aggregate of all the spatial weights:

$$S_{0}=\sum_{i=1}^{n}\sum_{j=1}^{n}w_{i,j}$$ (2)

The spatial weight calculation method adopted in this study is Queen, which has been frequently used [41,42]. In this calculation method, when two polygons have an edge overlap or a point overlap, they are considered to be adjacent, that is, marked as "1,"; otherwise marked as "0."

### Standard deviation ellipse

The Standard Deviational Ellipse (SDE) is given as follows [43,44]:

$$SDE=\left(\begin{array}{cc} \text{var}(x) & \text{cov}(x,y)\\ \text{cov}(y,x) & \text{var}(y) \end{array}\right)=\frac{1}{n}\left(\begin{array}{cc} \sum_{i=1}^{n}{\tilde{x_{i}}}^{2} & \sum_{i=1}^{n}\tilde{x_{i}}\tilde{y_{i}}\\ \sum_{i=1}^{n}\tilde{x_{i}}\tilde{y_{i}} & \sum_{i=1}^{n}{\tilde{y_{i}}}^{2} \end{array}\right)$$ (3)

where

$$\text{var}(x)=\frac{1}{n}\sum_{i=1}^{n}{\left(x_{i}-\bar{x}\right)}^{2}=\frac{1}{n}\sum_{i=1}^{n}{\tilde{x_{i}}}^{2}$$ (4)

$$\text{cov}(x,y)=\frac{1}{n}\sum_{i=1}^{n}\left(x_{i}-\bar{x})(y_{i}-\bar{y}\right)=\frac{1}{n}\sum_{i=1}^{n}\tilde{x_{i}}\tilde{y_{i}}$$ (5)

$$\text{var}(y)=\frac{1}{n}\sum_{i=1}^{n}{\left(y_{i}-\bar{y}\right)}^{2}=\frac{1}{n}\sum_{i=1}^{n}{\tilde{y_{i}}}^{2}$$ (6)

where *x*_*i*_ and *y*_*i*_ are the coordinates for feature *i*, $\left\{\bar{x},\bar{y}\right\}$ represents the mean center for the features, $\tilde{x_{i}}$ and $\tilde{y_{i}}$ represent the difference between *x*_*i*_ and $\bar{x}$, *y*_*i*_ and $\bar{y}$, respectively, and *n* is equal to the total number of features. In general, the center of the standard deviation ellipse expresses the spatial characteristics of the element agglomeration. When the centers of the standard deviation ellipses of different years have spatial displacements, the spatial agglomeration trend of this element has changed.

SDE describes the spatial pattern mainly in the direction, area and oblateness of the ellipse.

① Direction

The direction (*θ*) of the SDE is based on the X-axis and rotates clockwise at 0 degrees in the direction of true north (12 o’clock).

$$\text{tan}\;\theta =\frac{A+B}{\text{C}}$$ (7)

where

$$A=(\sum_{i=1}^{n}{\tilde{x_{i}}}^{2}-\sum_{i=1}^{n}{\tilde{y_{i}}}^{2})$$ (8)

$$B=\sqrt{{\left(\sum_{i=1}^{n}{\tilde{x_{i}}}^{2}-\sum_{i=1}^{n}{\tilde{y_{i}}}^{2}\right)}^{2}+4{\left(\sum_{i=1}^{n}\tilde{x_{i}}\tilde{y_{i}}\right)}^{2}}$$ (9)

$$C=2\sum_{i=1}^{n}\tilde{x_{i}}\tilde{y_{i}}$$ (10)

② Area

The area of SDE (*S*_*SDE*_) expresses the meaning of the distribution range of features.

$$S_{SDE}=\pi \phi_{x}\phi_{y}$$ (11)

where

$$\phi_{x}=\sqrt{2}\sqrt{\frac{\sum_{i=1}^{n}{\left(\tilde{x_{i}}\;\text{cos}\theta -\tilde{y_{i}}\;\text{sin}\theta \right)}^{2}}{n}}$$ (12)

$$\phi_{y}=\sqrt{2}\sqrt{\frac{\sum_{i=1}^{n}{\left(\tilde{x_{i}}\;\text{sin}\theta +\tilde{y_{i}}\;\text{cos}\theta \right)}^{2}}{n}}$$ (13)

③ Oblateness

The oblateness (*η*) of an ellipse reflects the degree of spatial clustering of data. The larger the oblateness, the stronger the centripetal force of the data; the smaller the oblateness, the weaker the centripetal force of the data.


$$\eta =\frac{\phi_{x}-\phi_{y}}{\phi_{x}}$$ (14)

In this paper, SDE is used to analyze the change in spatial patterns of the manufacturing industry. Notably, SDE is good at describing the overall spatial pattern of the data, whereas it relatively ignores the details of the spatial distribution of the data; however, this will not affect the support of SDE for research conclusions.

### Granger causality

The Granger causality test shows links between random variables by using empirical data sets and finding correlations between them [45]. A classical Granger causality test goes through the following flow: unit root test → cointegration test → vector autoregression (VAR) → Granger test.

For the unit root, the augmented Dickey-Fuller test (ADF) [46] was used in this study [47,48]. The ADF test fits a model of the form:

$$\Delta x_{t}=\mu +\gamma t+\alpha x_{t-1}+\sum_{j=1}^{k-1}\beta_{j}\Delta x_{t-j}+u_{t}$$ (15)

where *x*_*t*_ is a time series, Δ is the difference operator and *u*_*t*_ is a white-noise. *γ*, *α* and *β* are coefficients. *k*−1 is lag order. The test examines the negativity of the parameter *α* based on its regression *t* ratio.

In the cointegration test, we used the vector error-correction model (VECM), which is widely used in economic research [49–51]. An appropriate VECM model can be formulated as follows

$$\Delta y_{t}=\alpha \beta 'y_{t-1}+\sum_{t=1}^{p-1}\Gamma_{i}\Delta y_{t-i}+\omega_{t}$$ (16)

where **y** is a (*K*×1) vector of I(1) variables (a nonstationary process is integrated of order d, written I(d), if the process must be differenced d times to produce a stationary series), **α** and **β** are (*K*×*r*) parameter matrices with rank *r* < *K*, **Γ**_1_, **Γ**_2_,…,**Γ**_*p*−1_ are (*K*×*K*) matrices of parameters, and *ω*_*t*_ is vector residual.

The reliability of the VAR model is determined by checking eigenvalue stability. If the modulus of each eigenvalue of the matrix A is strictly less than one, the estimated VAR is stable [52,53]. The STATA can use the *varstable*,*graph* command to visually observe the stability of eigenvalues [54]. Finally, we can verify whether x is the Granger cause of y. A variable x is said to Granger-cause a variable y if, given the past values of y, past values of x are useful for predicting y. A common method for testing Granger causality is to regress y on its own and x lagged values and test the null hypothesis that the estimated coefficients on the lagged values of x are jointly zero. Failure to reject the null hypothesis is equivalent to failing to reject the hypothesis that x does not Granger-cause y [55]. Consider the following model

$$y_{t}=\psi +\sum_{m=1}^{p}\alpha_{m}y_{t-m}+\sum_{m=1}^{p}\beta_{m}x_{t-m}+\varepsilon_{t}$$ (17)

where *p* is the order of lag. Testing the null hypothesis *H*_0_ : *β*_1_ = ⋯ = *β*_*p*_ = 0 that the past value of *x* is not helpful in predicting the future value of *y*. If *H*_0_ is rejected, then *x* can be considered the Granger-cause of *y*.

### Data and area

The data used in this article are from the website Tianyancha (https://www.tianyancha.com/). On this website, we can query the establishment year, registered capital, and other information concerning manufacturing enterprises in various counties in Jiangsu. The index adopted in this paper is the registered capital of manufacturing enterprises. This strategy differs from those in existing studies that use employment numbers and output values to represent the development level of manufacturing industries. In addition, the raw data entries were broken down by company. Thus, we could further drill down into counties based on the participants’ addresses.

Based on the National Standard of the People’s Republic of China (GB/T 4754–2017), 29 manufacturing sectors were chosen for study in this paper. At the same time, we have organized the original data, such as by deleting duplicates.

Our research area, Jiangsu Province, is in China’s eastern coastal area. It has a developed economy. Since the administrative divisions of some counties in Jiangsu Province have been adjusted from 2000 to 2020, we have taken the administrative divisions of 2020 (96 counties) as the standard for this spatial research.

## Results

### Spatial autocorrelation analysis

**Fig 1:**
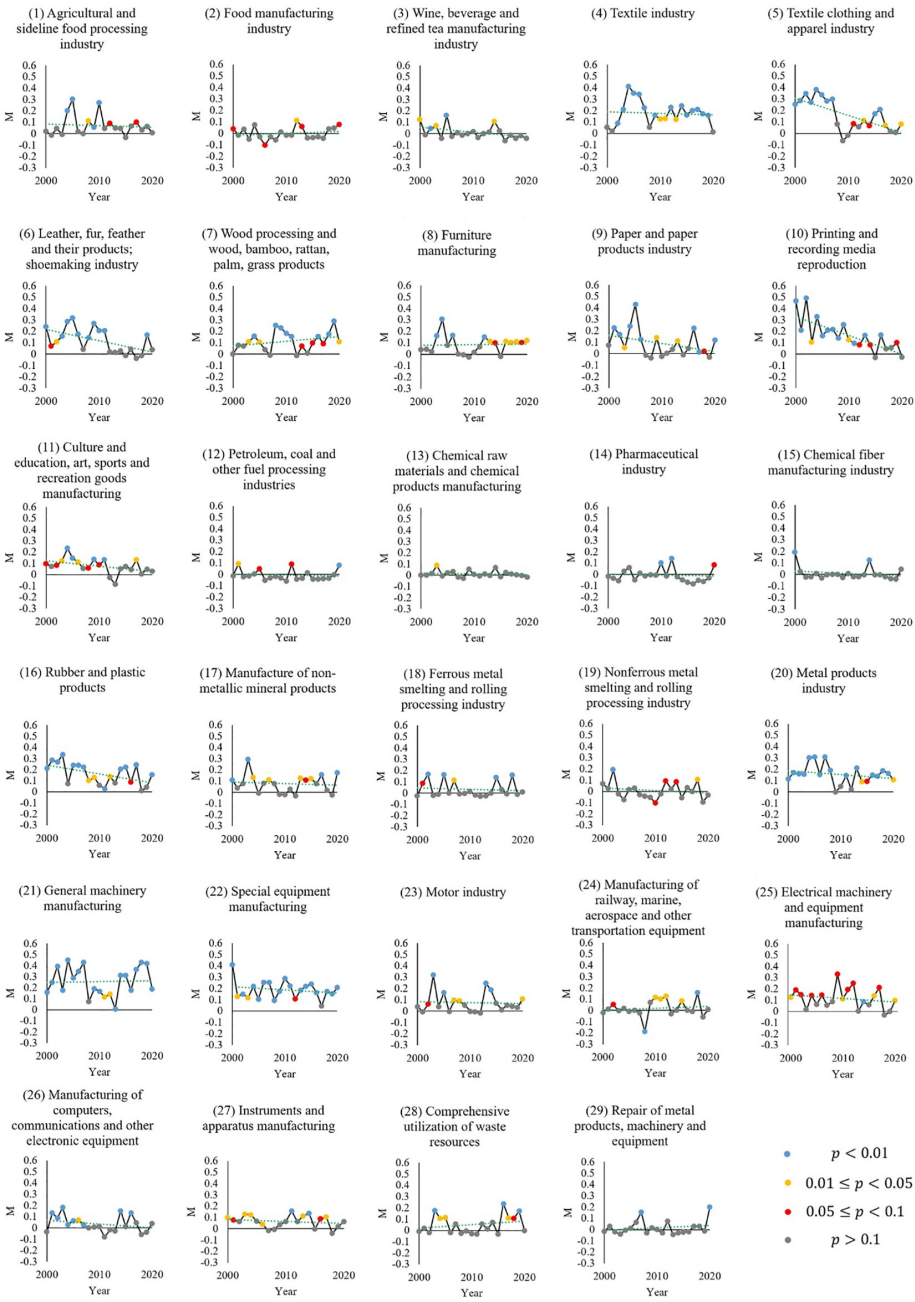
Moran’s I (M) of each manufacturing sector.

Fig 1 shows that each manufacturing industry type has a different significance level of M across several years. The most years with an M-value significance level below 0.1 occurred in: general machinery manufacturing, special equipment manufacturing, metal products industry, the textile industry, and printing and recording media reproduction. The spatial agglomeration of these manufacturing industries is highly obvious, forming economies of scale. However, the high concentration of the manufacturing industry has also caused environmental pollution in some areas of Jiangsu to a certain extent. Wang et al. argue that the level of the textile industry in Jiangsu Province is significantly correlated with the emission intensity of Chemical Oxygen Demand [56].

The manufacturing industries with the most years with an M-value significance level above 0.1 are chemical raw materials and products manufacturing, chemical fiber manufacturing industries, metal product repair, machinery, and equipment. At the same time, we can also see that when the *p*-value is high (>0.1), it frequently corresponds to a lower (or even negative) M-value. For example, the chemical fiber manufacturing industry between 2000 and 2020 experienced 18 years for which the *p*-value of the M-value was greater than 0.1. The M-value during these years also fluctuates around 0. These results indicate that the spatial distribution of some manufacturing industries is scattered. The scattered distribution has two possible explanations. First, these manufacturing industries did not form a relatively large growth pole at the early stage of development; second, as they grow, they have less need for agglomeration and economies of scale.

This gives the government inspiration. More specifically, for those manufacturing sectors with higher demand for agglomeration (especially green and low-carbon high-tech manufacturing), the government should create conditions conducive to agglomeration by improving the level of infrastructure such as transportation and communication and building a high-quality business environment. However, for the manufacturing industry with relatively low demand for agglomeration, the government can appropriately adopt more free and open management strategies. This can aid the government in using its limited financial funds and human resources more efficiently when promoting the development of local manufacturing industries.

On the other hand, regardless of the type of manufacturing, M-values fluctuate noticeably. In some manufacturing industries, the difference between the maximum and minimum values of M in the study period is substantial. For example, under the premise that p is less than 0.1, the differences in M-values between years are relatively large: 1) print and recording media reproduction: M_max_ = 0.49 (2002, p<0.01) and M_min_ = 0.08 (2014, p<0.1); 2) the paper and paper products industry: M_max_ = 0.43 (2005, p<0.01) and M_min_ = 0.02 (2017, p<0.01); 3) the textile clothing and apparel industry: M_max_ = 0.38 (2004, p<0.01) and M_min_ = 0.07 (2014, p<0.1).

**Fig 2:**
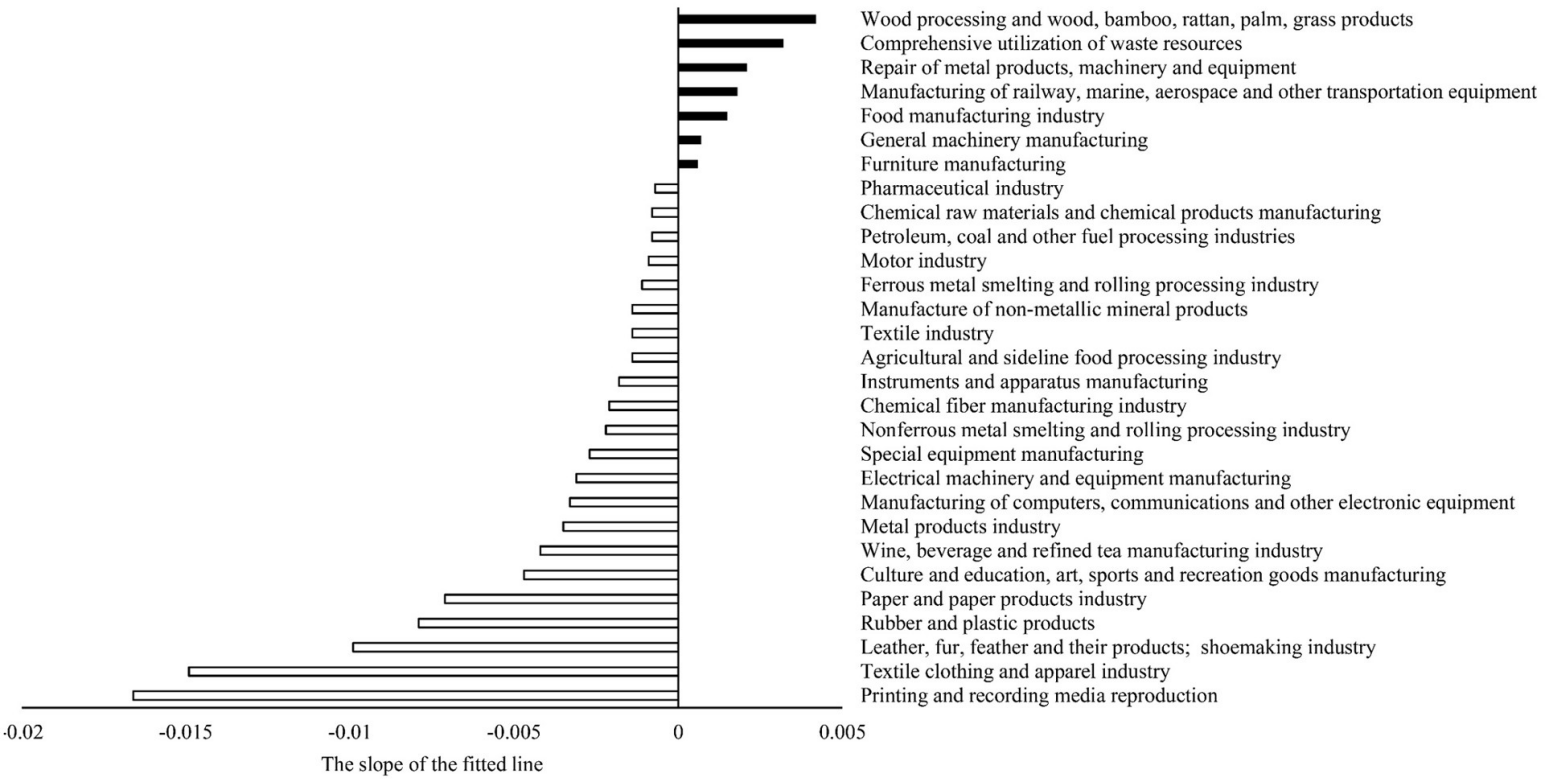
The slope of the fitted line for the changing trend of M of each manufacturing sector over the years.

This volatility in many manufacturing subcategories is reflected in an overall downward trend in M-value. We performed linear fitting for the interannual M-value changes for all manufacturing industry types (Fig 1) and calculated the slopes of each fitting line respectively. Fig 2 shows the slopes arranged from largest to smallest.

Fig 2 shows a downward interannual change trend of M-values for 22 manufacturing categories (the slope of the fitted line is negative). In contrast, the interannual change trend of M of 7 manufacturing categories is upward (the slope of the fitted line is positive). This phenomenon indicates that the degree of spatial autocorrelation of registered capital is decreasing for most manufacturing industries in Jiangsu Province. New manufacturers increasingly disregard what was once considered more commercially logical: the idea that they should be physically closer to each other.

**Fig 3:**
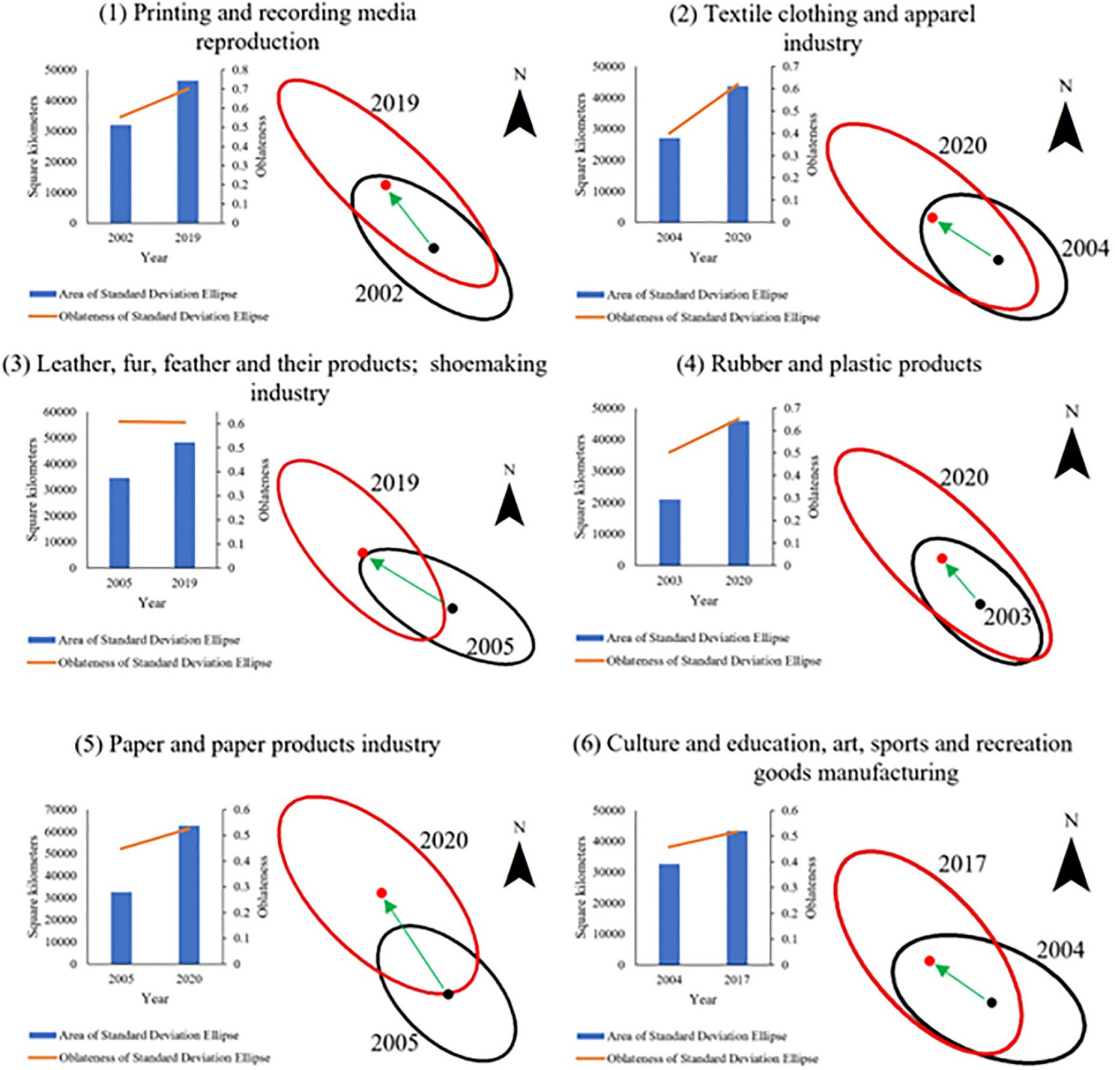
The SDE of six manufacturing sectors.

When we use the SDE for visualization, we more clearly see the transformation of some manufacturing industry types from “relatively concentrated” to “relatively dispersed” in space. Fig 3 shows the trajectory of the six manufacturing industries with the most significant decline in M (p<0.1) from 2000 to 2020. We selected the years with the maximum and minimum M-values. Then, we calculated the SDE for these two years. The spatial transfer (the change of center point position of the SDE) of these six manufacturing industries indicates an obvious direction: from south to north. From black SDE to red SDE in six manufacturing sectors, the area of SDE increased by the following proportions: 45.17% (Printing and recording media reproduction), 61.66% (Textile clothing and apparel industry), 39.27% (Leather, fur, feather and their products; shoemaking industry), 118.53% (Rubber and plastic products), 92.59% (Paper and paper products industry), and 33.18% (Culture and education, art, sports and recreation goods manufacturing).

The increase in the area of SDE indicates the expansion trend in the spatial coverage of these six manufacturing industries. This shows that northern Jiangsu is becoming a central area for manufacturing enterprises to invest in. The increase in oblateness also coincides with the finding that as manufacturing expands from south to north, SDE becomes more directional, or more specifically, thinner and longer. For instance, the SDE’s oblateness of the textile clothing and apparel industry increased from 0.40 to 0.62. The transfer of manufacturing from south to north is closely related to the Jiangsu government. One study argues that this is related to the Jiangsu government’s deployment of the Central Committee of the Communist Party of China and The State Council on high-quality development and regional coordinated development [57].

In fact, since the beginning of this century, the people’s Government of Jiangsu Province has begun to implement the “North—South Pairing” plan, constructing the “South helps North” program between Suzhou and Suqian, Wuxi and Xuzhou, Nanjing and Huaian, Changzhou and Yancheng, Zhenjiang and Lianyungang. We take Suzhou and Suqian as an example to analyze the impact of this scheme on the expansion of manufacturing from south to north. In order to help Suqian develop its manufacturing industry, in November 2006, the two cities jointly established the “Suzhou Suqian Industrial Park (SSIP),” which is located in Sucheng District of Suqian City. In the case of Sucheng District, among the 22 manufacturing categories with an overall spatial diffusion trend (see Fig 2, that is, the fitting slope is less than 0), 71.43% of the manufacturing sectors had an average annual registered capital growth rate from 2007 to 2009 that was higher than that from 2004 to 2006 (some manufacturing sectors had zero registered capital in some years, which could not be used as the denominator to calculate the growth rate, so they were omitted). That is, after the establishment of the SSIP, the registered capital of the manufacturing sector increased significantly. This is the result of the joint efforts of the Jiangsu provincial government, the Suzhou municipal government, and the Suqian municipal government.

However, the relocation of manufacturing has also caused environmental pressure in northern Jiangsu [58], which must be considered by the government when formulating relevant policies and planning schemes.

### Analysis of power law distribution

Thousands of new businesses have been established across the province for all manufacturing types. The registered capital amounts of these new enterprises vary considerably. For some, it exceeds 10 million yuan; for others, it is only tens of thousands. If we were to rank the registered capital of each new business in each manufacturing industry annually in descending order, we would get a curve with a steep head and a long, flat tail. Such curves generally conform to a power law distribution with the following formula:

$$c=a\cdot r^{b}$$ (18)

Here, *c* stands for registered capital; *r* represents the sequence number in descending order; *a* and *b* are parameters. Generally, the larger the absolute value of *b* is, the higher the data value is that is only occupied by a few observation points. The observation point values with high proportions in the total number are minimal.

**Fig 4:**
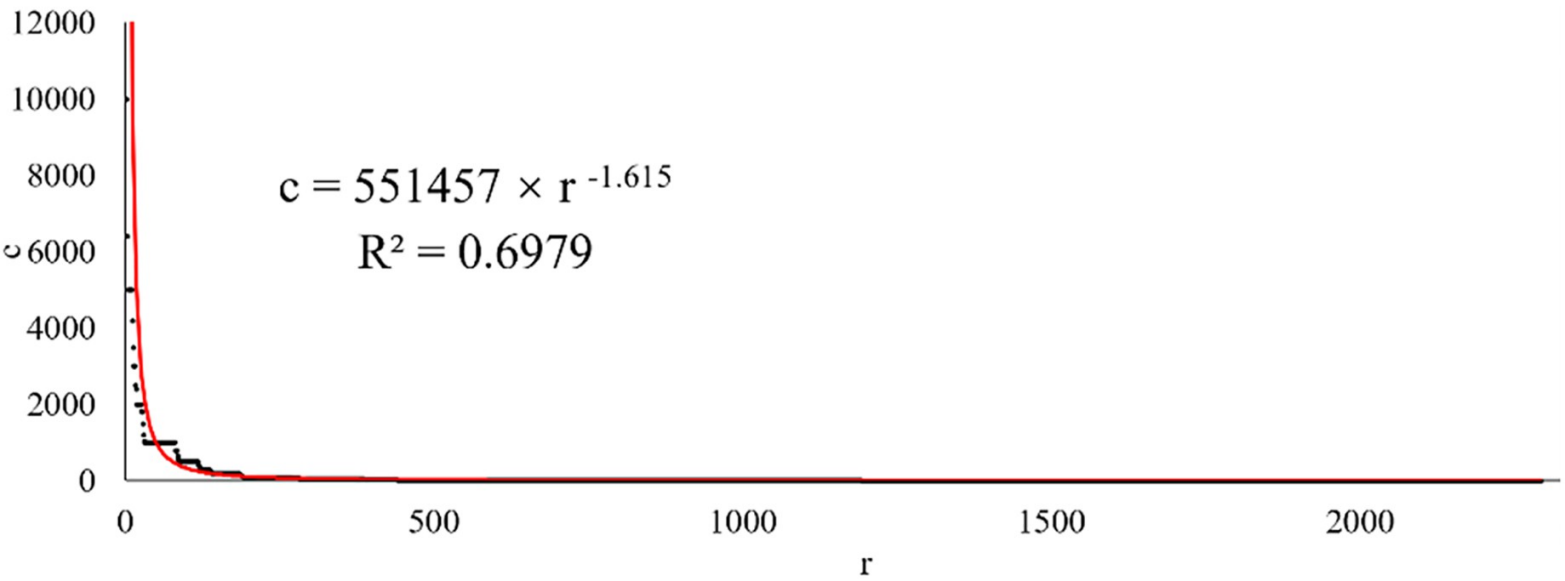
The descending order of registered capital of new enterprises in the agricultural and sideline food processing industry with a fitting curve (2020).

For example, the agricultural and sideline food processing industry illustrates the characteristics of registered capital amounts of new enterprises in manufacturing. Fig 4 shows the scatter plot and fitting curve of all new 2020 enterprises in this manufacturing industry in descending order of registered capital. The power law characteristics of the fitting curve are apparent. In addition, the absolute value of *b* in the fitting formula reaches 1.615. This result shows that most new enterprises’ registered capital amount is low.

Two questions readily come to mind. First, how does the *b* of the same manufacturing industry change annually? Second, how do the *b*-values of different manufacturing industries differ? The chart below shows an analysis of these problems.

From this graph, we quickly arrive at the following findings: (1) In most manufacturing industries during 2000–2020, at some point, *b* was significantly smaller for at least about 2–3 years, showing several obvious downward “spikes” in the graph. (2) In most manufacturing industries, the change range of *b* was stable in most years. Furthermore, the changing trend in *b* neither rose nor declined overall during 2000–2020. (3) The value of *b* in different manufacturing industries may vary significantly.

We may ask why some manufacturing industries “spike” in certain years. The absolute value of *b* is significantly greater in some years than others. These years saw the creation of large new companies with far more registered capital than others. For example, the textile clothing and apparel industry had 6,392 newly established companies in Jiangsu Province in 2009. Among these new companies, the highest registered capital was 5 billion yuan. The second- to fifth-ranked registered capital amounts were 300 million, 221 million, 218 million, and 102 million yuan. All of these values significantly differed from 5 billion yuan.

**Fig 5:**
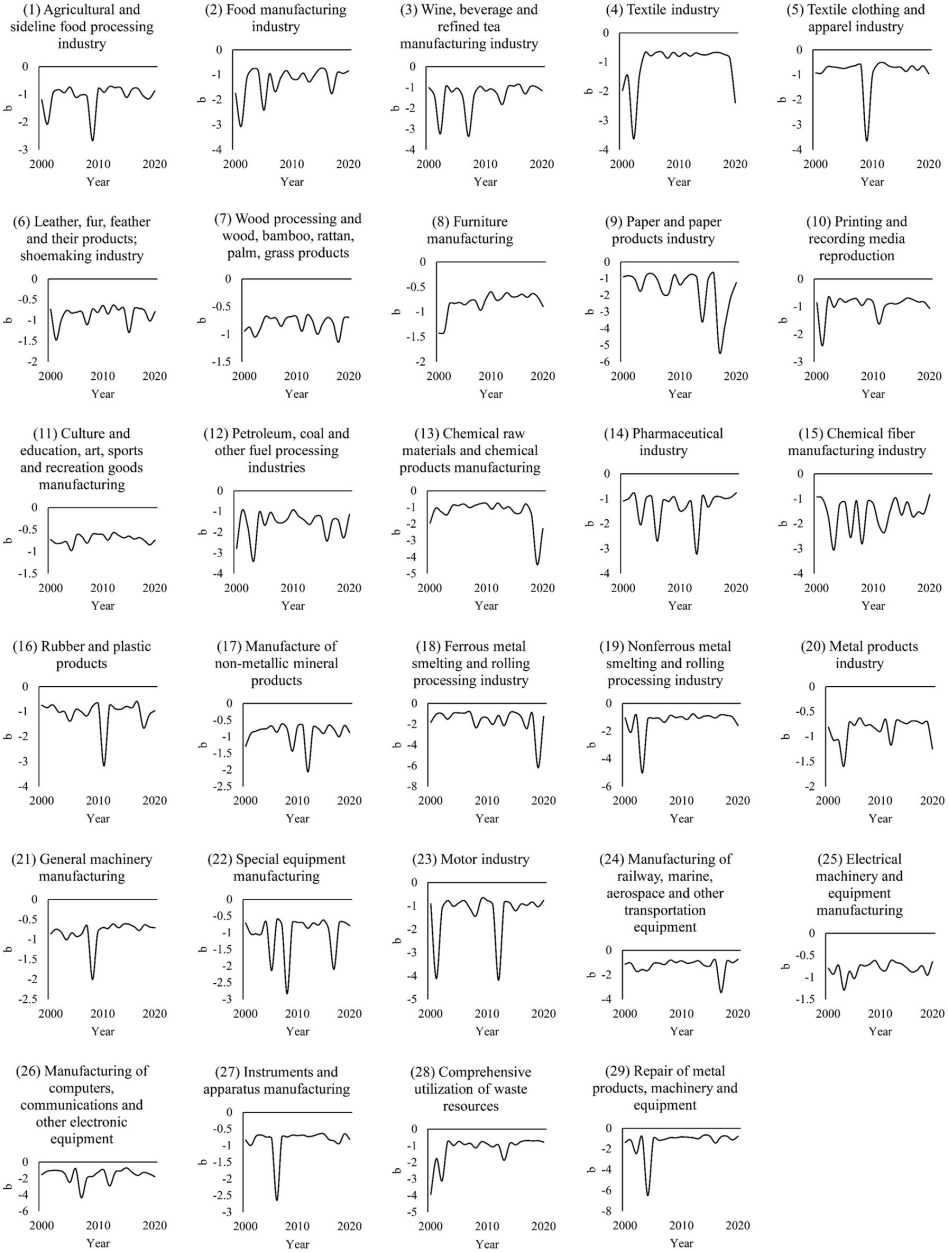
Different years of *b* in various manufacturing industries.

The *b*-value stabilizes within a range in most years because, in each manufacturing industry, the proportion of firms of all sizes in its market is relatively constant. In other words, the leading firms are large, but their numbers are few. In contrast, the smallest firms are plentiful. However, this polarization is subtle in some industries, such as in culture and education, art, sports and recreation goods, and furniture manufacturing. Fig 5 shows that the absolute value of *b* for these manufacturing industries has remained below 1 for years. Compared with heavy and chemical industries requiring substantial capital investment, these are generally smaller in scale and volume.

This type of power law distribution in size and quantity is common in economic research. Therefore, the government should formulate policies in a targeted manner. For example, for several small manufacturing enterprises, the government should give them preferential policies considering the tax revenue they can bring to the local government and the jobs they can provide. For several small-scale manufacturing enterprises, the government should provide support measures, such as business strategy guidance, small low-interest loans, and technology exchange platforms.

### Analysis of the scatterplot of M and b: The non-agglomeration tendency of large enterprises

**Fig 6:**
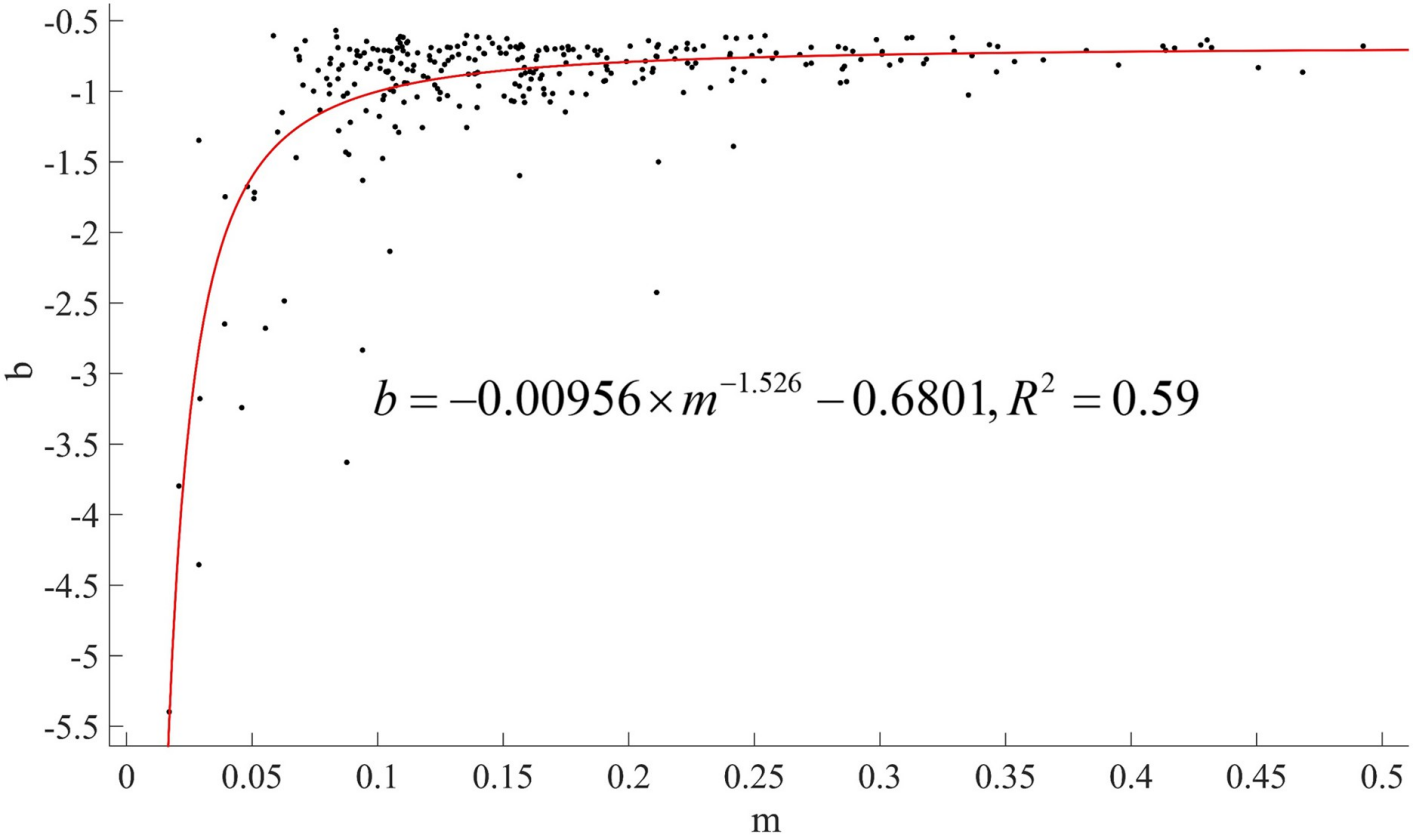
A scatterplot of M and *b*.

When we put M and *b* together, something interesting happens. First, we select the M (excluding *p*-values above 0.1) for each manufacturing sector in different years. Then, we plot M and *b* of the same year and the manufacturing sector as a point’s horizontal and vertical coordinates. Finally, we fit these scatter points. As M increases, *b* at first rises rapidly. However, when M exceeds a specific value (about 0.1), the upward momentum of *b* slows significantly as M increases (Fig 6).

When *b* is small (large in absolute value), one or several new enterprises with substantial registered capital were established in this manufacturing sector in the year. In this case, M is relatively small. Therefore, the spatial autocorrelation degree of newly added registered capital of this manufacturing sector in this year is low. This value indicates that those large enterprises with abundant financial resources did not cluster when incorporated. These clusters tend to comprise many enterprises. However, they have small scales of registered capital within single enterprises.

**Fig 7:**
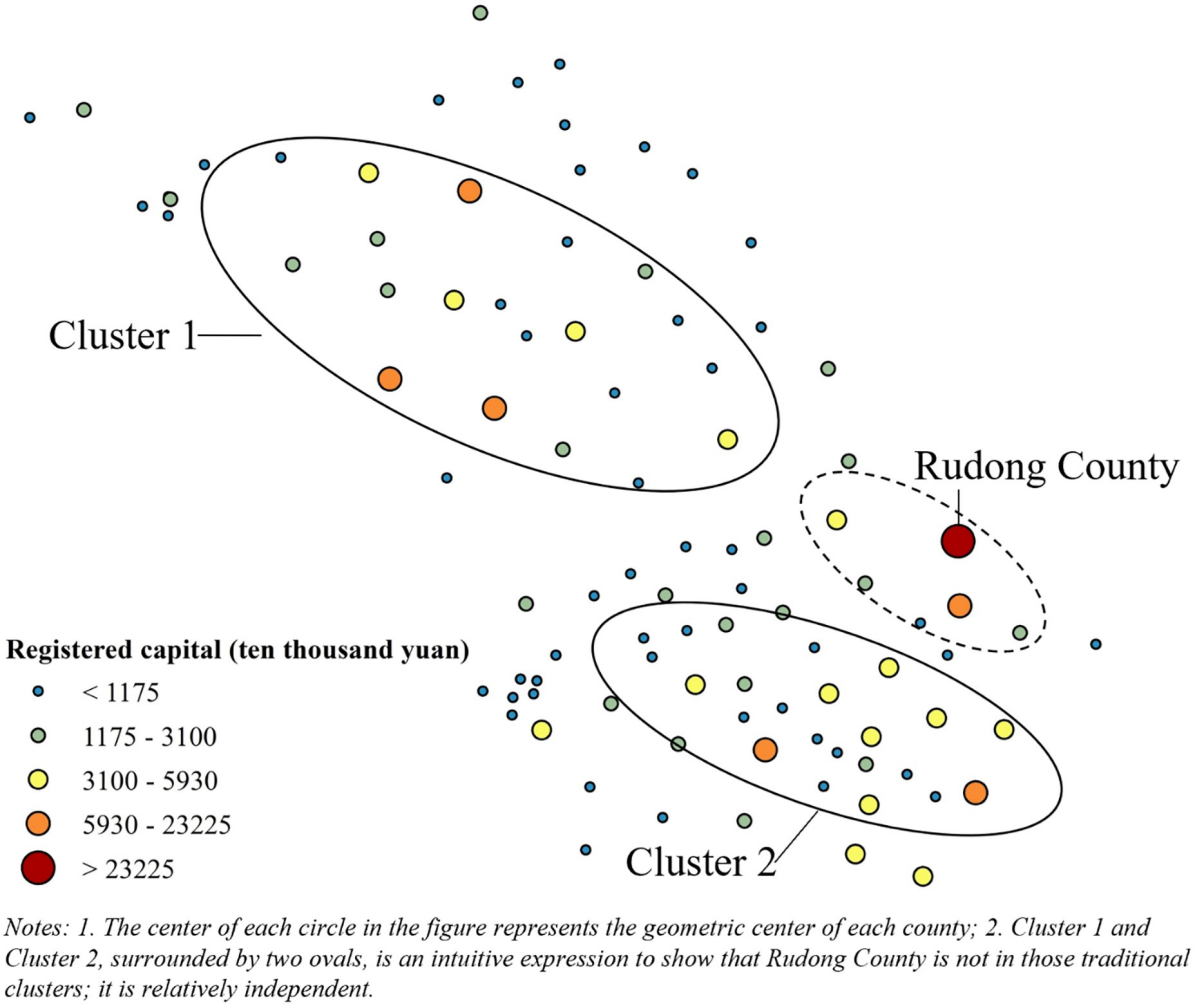
The newly registered capital of each county of the paper and paper products industry in 2018.

A suitable example is the 2018 paper and paper products industry. Fig 7 shows this sector’s newly added registered capital by counties in Jiangsu that year. The county with the highest registered capital is Rudong County. However, it does not lie within Clusters 1 or 2. Rudong has the highest registered capital because one large company (Jinhuasheng Paper [Nantong] Co., LTD) invested 2.298 billion yuan in the county to establish new factories in 2018.

The “non-agglomeration” tendency of large companies when investing in opening new factories is reflected in the above example. It is also relatively common throughout Jiangsu Province. These tendencies demonstrate that large companies have more capital to develop. Therefore, they can do more of the work independently. In contrast, smaller companies often need to pool resources and work together.

### The relationship between spatial agglomeration characteristics of the manufacturing industry and regional economic differences

#### Regional economy differences in Jiangsu Province

GDP Per capita (GDPPC) is an important index to measure the degree of economic development of a city. The coefficient of variation (CV) between the GDPPC of different cities within a province can be used to measure economic differences within a province.

**Fig 8:**
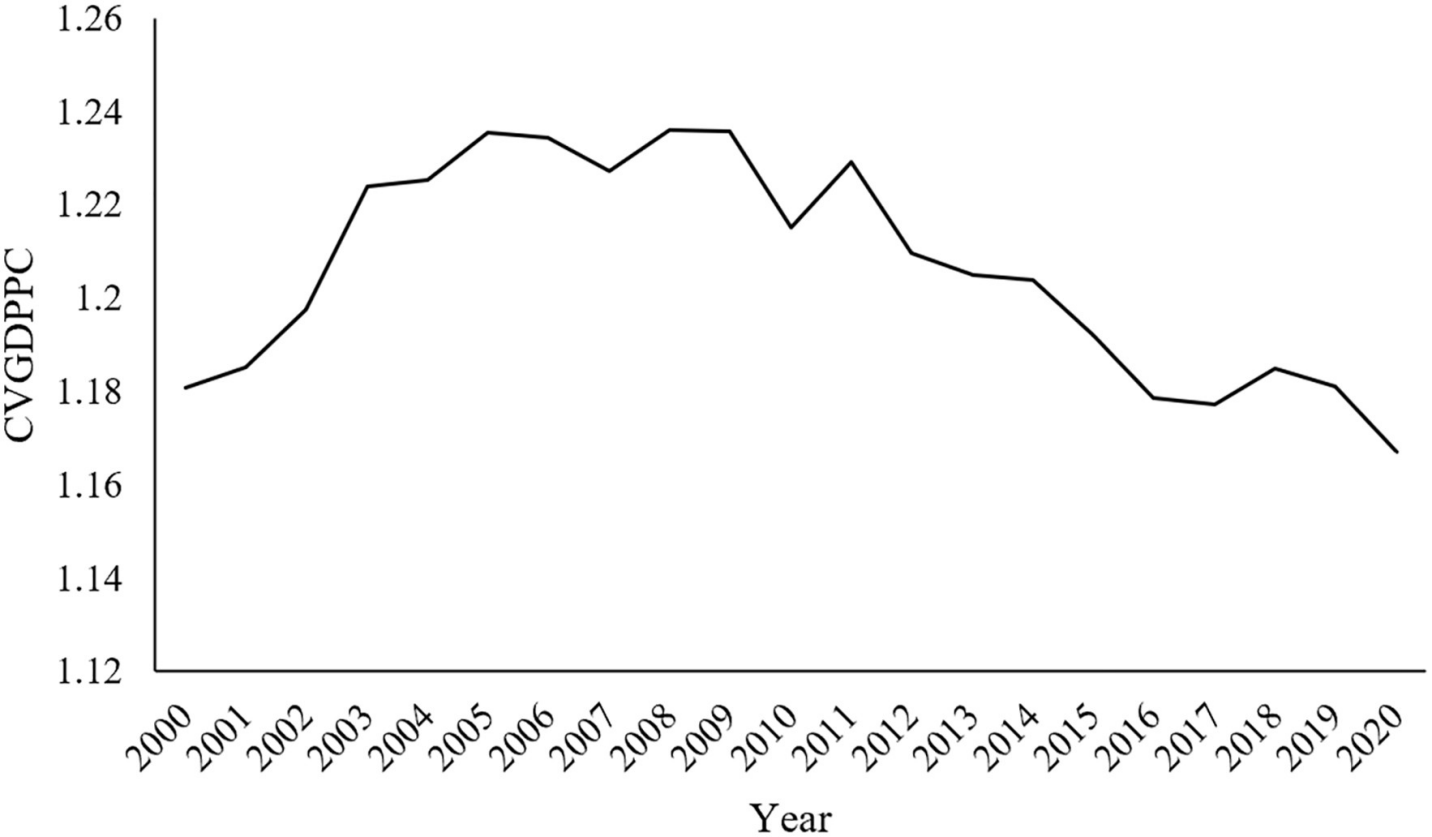
CVGDPPC of 13 cities in Jiangsu Province in different years.

As shown in Fig 8, the CV of GDPPC (CVGDPPC) first increases and then decreases during 2000–2020. This shows that the economic differences between different regions in Jiangsu Province have experienced a process of expansion followed by a reduction.

#### M average values for 29 manufacturing sectors

**Fig 9:**
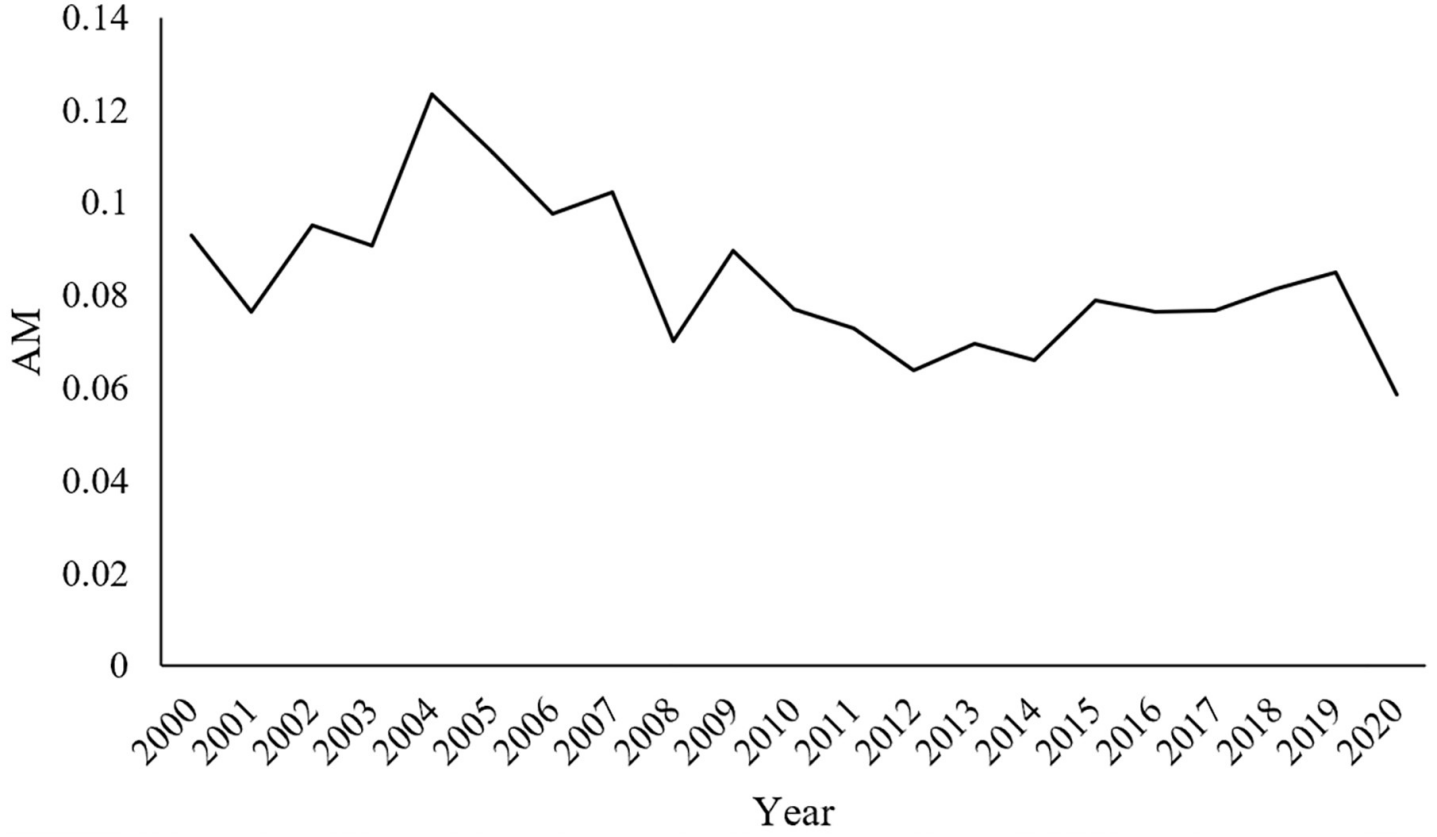
M average values for 29 manufacturing sectors.

The average value of M (AM) for the 29 manufacturing sectors shows a downward trend between 2000 and 2020, which is consistent with the above analysis—that is, the spatial agglomeration of the manufacturing industry in Jiangsu Province is weakening (Fig 9).

#### An analysis of the relationship between AM and CVGDPPC based on the Granger causality test

An empirical analysis of the relationship between the AM and the CVGDPPC was conducted. When a second-order difference was made for AM and CVGDPPC, both passed the unit root test (Table 1). Furthermore, after a lag of four orders, results showed a cointegration relationship (Table 2).

**Table 1:** Unit root test results.

		Test statistic	Dickey-Fuller critical value			MacKinnon approximate p-value for Z(t)
			1%	5%	10%	
**AM**	**z(t)**	-10.653	-3.750	-3.000	-2.630	0
**CVGDPPC**	**z(t)**	-7.599	-3.750	-3.000	-2.630	0

Note: The meaning of z(t) is t statistic (This statistic is applicable to the relatively small sample size and is one of the most important indicators in the unit root test.); Dickey-Fuller Critical Values are a statistic used to test whether time series data has a unit root. In this test, we can see that both “-10.653” and “-7.599” are less than the critical value at the 1% level (-3.75), so the sequence is stationary. The MacKinnon approximate p-value for Z(t) is equal to 0, less than 0.01, so it also supports, from another perspective, that the time series is stationary.

**Table 2:** Cointegration test results.

Maximum rank	Params	LL	Eigenvalue	Trace statistic	Critical value 5%
**0**	14	92.3449		18.0458	15.41
**1**	17	100.0368	0.6414	2.6621*	3.76
**2**	18	101.3678	0.1626		

Note: The values 0, 1, and 2 of Maximum rank indicate no cointegration relationship, one cointegration at most, and two cointegrations at most, respectively. Under the Johansen cointegration test adopted in this study, for the hypothesis of “no cointegration relationship,” the trace statistic value is 18.0458, which is greater than the critical value of 15.41 under 5%, so the hypothesis should be rejected, indicating that there is at least one cointegration relationship. For the hypothesis of “at most one cointegration,” the value of the trace statistic is 2.6621, which is less than the critical value of 3.76 and under 5%. This hypothesis should be accepted, indicating that there is no more than one cointegration relationship. Therefore, it follows that there is a cointegration relationship between the two variables. This tells us which rank should be selected. LL is Log Likelihood.

Regarding the VAR model, according to Table 3, the optimal lag order of the VAR model is 2. This is because AIC, HQIC, and SBIC all have a lag equal to 2 (with “*”).

**Table 3:** Determination of the optimal lag order of VAR model.

Lag	LL	LR	df	p	FPE	AIC	HQIC	SBIC
**0**	76.5097				1.7×10^−7^	-9.93463	-9.93563	-9.84022
**1**	85.1696	17.32	4	0.002	9.0×10^−8^	-10.5559	-10.559	-10.2727
**2**	94.1666	17.994	4	0.001	4.8×10^−8^	-11.2222*	-11.2272*	-10.7502*
**3**	96.2579	4.1827	4	0.382	6.9×10^−8^	-10.9677	-10.9748	-10.3069
**4**	101.368	10.22*	4	0.037	7.4×10^−8^	-11.1157	-11.1248	-10.266

Note: LL is Log Likelihood; LR is Likelihood Ratio; df is degrees of freedom; FPE is Final Prediction Error; AIC is Akaike Information Criterion; HQIC is Hannan—Quinn Information Criterion; SBIC is Schwarz Bayesian Information Criterion.

**Fig 10:**
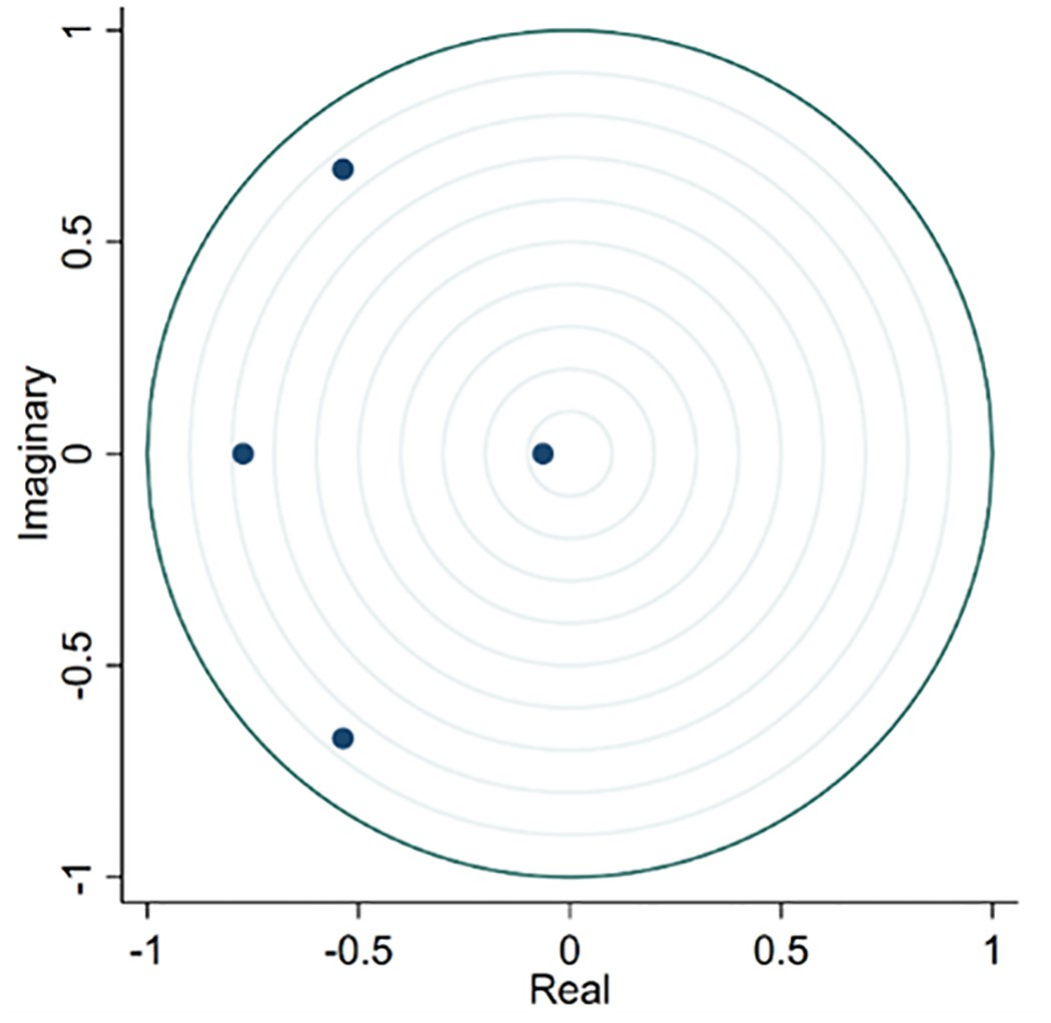
Roots of the companion matrix.

Furthermore, Fig 10 shows that all eigenvalues fall within the circle; therefore, this VAR model is stable.

Table 4 shows the results of the Granger causality test. We cannot reject the hypothesis that ddAM does not Granger-cause ddCVGDPPC. However, we can reject the hypothesis that ddCVGDPPC does not Granger-cause ddAM. These show that CVGDPPC after the second-order difference is the Granger cause of the AM after the second-order difference.

**Table 4:** Granger causality test results.

Equation	Excluded	chi2	df	Prob>chi2
**ddCVGDPPC**	ddAM	1.8082	2	0.405
**ddCVGDPPC**	ALL	1.8082	2	0.405
**ddAM**	ddCVGDPPC	10.37	2	0.006
**ddAM**	ALL	10.37	2	0.006

Note: ddCVGDPPC indicates the CVGDPPC after second-order difference; ddAM indicates the AM after second-order difference; Chi2 is Wald Chi-squared test; df is degree of freedom.

**Fig 11:**
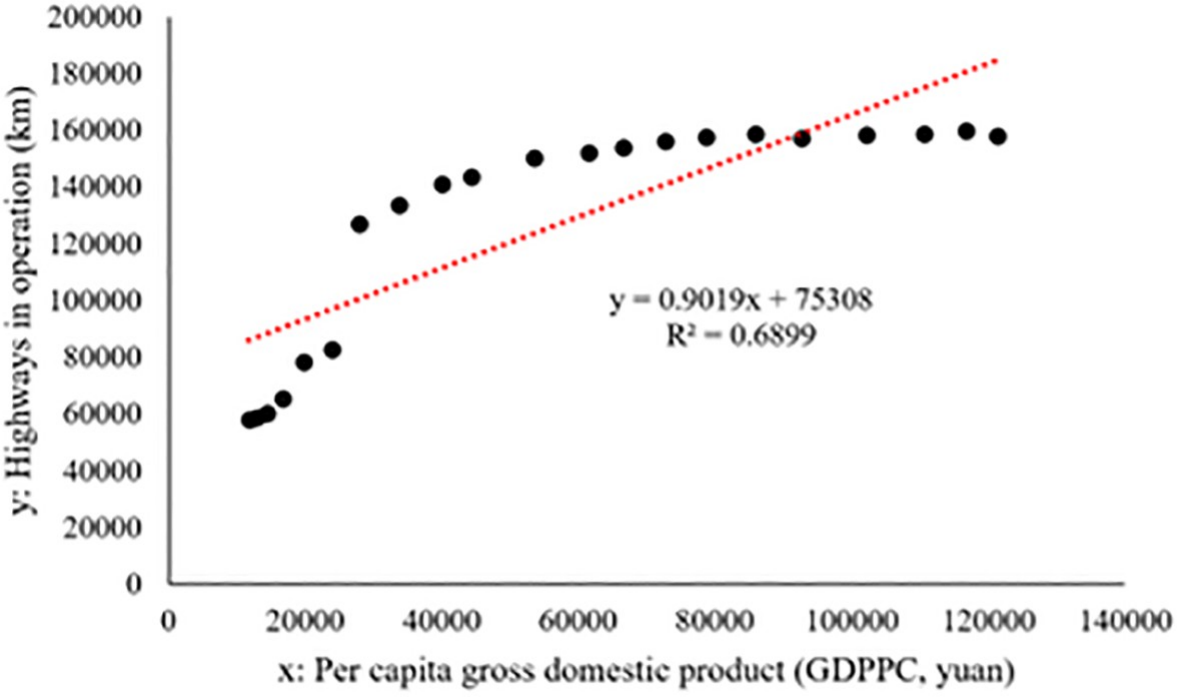
GDPPC and highways in operation in Jiangsu Province from 2000–2020.

These analyses have proven that the reduction of regional economic differences affects the spatial distribution of manufacturing industry to some extent. The essence of the reduction of regional economic difference in Jiangsu is the rapid economic development of northern Jiangsu and central Jiangsu. With this rapid development, transportation facilities and other positive factors that provide support for the development of the manufacturing industry continue to increase (as we show in Fig 11, as the economy grows, so does the number of road miles), attracting more and more manufacturing sectors to invest in northern and central Jiangsu.

## Discussion

In the early years of this century, most manufacturing enterprises preferred southern counties with better economic bases when they invested in production. Moreover, the demand for spatial agglomeration for sharing infrastructure and business information was significant for earlier manufacturing enterprises. These concerns were constrained by communication and transportation conditions far less developed than today.

However, this dependence on agglomeration slowly changed over our study period. Many counties, especially those in northern Jiangsu Province, enjoy continuously improving development conditions, including more convenient transportation (highways, high-speed railways), smoother information networks, and more efficient organization of government and management. Thus, many manufacturing enterprises are now more willing to invest in these areas because the relatively low industrial concentration in the region leads to lower rents and a more urgent need for urbanization and industrialization. The governments of China and Jiangsu Province have attached great importance to the province’s balanced economic development. This interest has directly guided the change of the manufacturing industry’s spatial layout.

In the vital economic and historical process of evolving manufacturing patterns, large enterprises invest in and build factories in regions with relatively low industrial agglomeration. They can do so because of their greater financial strength and ability to protect against risk and provide substantial employment opportunities. Government officials in less-developed counties, especially those in charge of industrial and commercial management, are more than willing to convince large manufacturers to invest in their county.

Although the Granger test proved that the reduction of regional economic differences in Jiangsu Province is related to the spatial diffusion of the manufacturing industry, at least two topics require the attention of future research. First, what will the relocation and diffusion of manufacturing bring to the local area? For example, how will it affect local employment and finance? Second, does the improvement of infrastructure conditions brought about by economic development have different mechanisms for the transfer and diffusion of different manufacturing sectors? These problems are worthy of further study.

The Jiangsu manufacturing industry’s development plays a significant role in the provincial economy. Thus, its contribution to China’s economic growth cannot be ignored. Therefore, the research on relevant issues in this paper may, to a certain extent, provide referential countermeasures and suggestions for the future transformation and upgrading of Jiangsu Province’s manufacturing industry.

This study differs from previous studies as follows: First, we analyzed the changing spatial patterns of multiple manufacturing industries, whereas many previous insightful studies have focused more on one type or category of manufacturing such as advanced manufacturing [59], and the instrument-manufacturing industry [60]. Different types of manufacturing industries do exhibit different characteristics in their spatial distribution. Second, we effectively correlated the intrinsic structure of registered capital with the spatial pattern, which in turn, revealed, that while the manufacturing industry as a whole exhibits the characteristics of spatial agglomeration, some of the larger firms have chosen to move moderately away from the center.

However, this article still has some limitations. For example, because of the need for more microscopic interview data, we did not analyze why those large companies chose places with fewer industrial clusters from the business operations perspective. Our research team will continue to expand on this in subsequent related studies.

## Conclusion

The present study used spatial analysis to investigate the spatial-temporal pattern evolution of capital injection by different manufacturing sectors in Jiangsu Province during 2000–2020. Its main conclusions are as follows.

The spatial autocorrelation of most manufacturing sectors shows a downward trend when investing in plant construction. An increasing number of enterprises no longer regard gathering in the developed areas of southern Jiangsu as a vital investment principle. As a result, many departments are expanding from southern to northern Jiangsu.

The registered capital of most enterprises is similar. The number of top enterprises with large capital injection accounts for a minuscule proportion of the total manufacturing enterprises.

When investing in and building factories, many large enterprises tend not to cluster with numerous small enterprises in an industrial agglomeration area. Instead, they prefer areas with relatively low degrees of agglomeration.

The economic development of northern and central Jiangsu has narrowed the gap with southern Jiangsu and brought better infrastructure conditions, which is conducive to attracting more manufacturing sectors to invest there.

Therefore, this study has some insights for both governments and business. For the government, policymakers should carefully formulate some supportive policies or plans to help companies that choose to invest in northern Jiangsu to achieve better local economic growth. Enterprises should choose their own development paths according to scale to maximize their business interests.

## Supporting information

S1 File Moran’s I. (XLSX)

S2 File Data used to calculate SDE. (XLSX)

S3 File Registered capital of new enterprises in the agricultural and sideline food processing industry in 2020. (XLSX)

S4 File Value of b. (XLSX)

S5 File The newly registered capital of each county of the paper and paper products industry in 2018. (XLSX)

S6 File CVGDPPC. (XLSX)

S7 File M average values for 29 manufacturing sectors. (XLSX)

S8 File GDPPC and highways in operation in Jiangsu Province. (XLSX)
